# Physiological and Biochemical Responses to Repeated Administration of Ivermectin in Rabbits

**DOI:** 10.12688/f1000research.174211.1

**Published:** 2026-02-10

**Authors:** Mustafa A. Saud, Sabea Khamees Abed, Mohammed Ali Shahooth, Mohammad Jadaan, Ahmed Emad Abood

**Affiliations:** 1 College of Veterinary Medicine, University of Fallujah, Al-Fallujah, Al Anbar Governorate, 00964, Iraq; 2 Anatomy, University of Anbar, Ramadi, Al Anbar Governorate, Iraq

**Keywords:** Ivermectin, Biochemical, Electrolytes, Oxidative Markers

## Abstract

**Background:**

While ivermectin is a common antiparasitic drug used in veterinary medicine, the effects of repeated administration at therapeutic doses have not been well studied. Of particular interest is the impact of ivermectin on electrolyte balance, blood biochemistry, and oxidation-related markers in rabbits, as these animals are known to be sensitive to drug-related metabolic disturbances and side effects.

**Methods:**

A total of 10 clinically healthy adult male rabbits (1.5-2 kg) were randomly assigned to a control group with no treatment or to a treatment group receiving ivermectin subcutaneously at a dose of 0.25 mg/kg once weekly for 30 days. The animals were housed in the physiological laboratory of the University of Fallujah under controlled environmental conditions. At the end of the experiment, blood samples were collected, and serum was analyzed for electrolytes, biochemical and oxidative markers, and liver enzyme activity using established laboratory methods.

**Results:**

Repeated administration of ivermectin caused a statistically significant increase in potassium, sodium, chloride, and phosphate concentrations (P≤0.05) compared with baseline, while calcium and magnesium levels remained unchanged. Glucose, total protein, creatinine, cholesterol, and triglyceride levels increased statistically significantly (P≤0.05) compared with the control group, while albumin concentration decreased. Oxidative stress indicators showed increased levels of malondialdehyde (MDA) and decreased levels of reduced glutathione (GSH), as well as superoxide dismutase (SOD) activity. Increased alanine aminotransferase (ALT) and aspartate aminotransferase (AST) activity were also noted.

**Conclusions:**

Repeated administration of ivermectin at therapeutic doses at short intervals causes disturbance in blood electrolyte balance, biochemical profile, and antioxidant system in rabbits, and may also negatively impact liver and kidney functions. These results indicate the need for cautions use of the drug, preferably limited to single administration at long intervals, to minimize the risk of side effects.

## Introduction

Ivermectin is a semisynthetic derivative of a vermectin, a naturally occurring macrocyclic lactone produced by the bacterium *Streptomyces avermitilus* ( Hazan, 2022). This lipophilic endotoxin exhibits broad -spectrum antiparasitic activity and is widely employed in both veterinary and human health managements to control nematodes, arthropods, and other parasitic infections ( Hasan et al., 2022). Ivermectin is commonly used in animal husbandry systems for the prevention and treatment of infections caused by internal parasites in horses, cattle, sheep and goats ( Parisi et al., 2019; Bordes et al., 2020; Ahmed et al., 2020). Due to its wide use, the physiological and pharmacological effects of ivermectin on target species remain the subject of ongoing research. Ivermectin is the only avermectin derivative authorised for use in humans and is effective against parasitic infections such as onchocerciasis and lymphatic filariasis. Its pharmacological action is thought to be mainly mediated by modulation of glutamate-gated chloride channels in invertebrate nerve and muscle cells, resulting in increased chloride flow, hyperpolarisation of the membrane, paralysis and ultimately death of the parasites ( Wolstenholme and Neveu, 2022).

Importantly, ivermectin has a broad margin of safety in mammals, since these channels are not present in vertebrate neurons and its effect on mammalian (gamma-aminobutyric acid-mediated chloride) channels at therapeutic doses is negligible ( Shwaish et al., 2024). Despite its proven efficacy and extensive therapeutic use, there were concerns about possible adverse reactions in treated animals. Ivermectin exhibits a variety of pharmacokinetic properties, including high solubility of the lipid, long half-life and extensive tissue distribution, especially in the liver and adipose tissue ( Hennessy and Alvinerie, 2002). These properties may cause bioaccumulation and delayed elimination, especially via milk excretion during lactation. Ivermectin is also extensively metabolised in the liver by cytochrome P450 (CYP450) enzymes. Experimental studies indicate that ivermectin and its metabolites may inhibit the cytochrome P450 system and related drug transporters in mammals, potentially resulting in hepatocyte damage, metabolic abnormalities and immunotoxic or nephrotoxic effects ( Salman et al., 2022).

Several studies have shown that avermectins (ivermectin, abamectin, doramectin and eprinomectin) may cause a variety of biochemical, hormonal and histopathological changes in animals exposed to these substances ( GabAllh et al., 2017; Salman et al., 2022). In cattle, prolonged or repeated dosing of these substances, even at therapeutic levels, has been associated with disturbances of endocrine function and reproduction physiology. This includes dysregulation such as hormonal imbalances, altered reproductive patterns and reduced fertility ( Sadek and Shaheen, 2015; Nicolas et al., 2020). In view of these observations, further investigation is needed to determine whether ivermectin alters physiological homeostasis. Changes in serum biochemistry, electrolyte balance and oxidative stress parameters may provide critical information on the systemic effects of a pharmacologically active substance and the mechanisms underlying adverse reactions. Based on this, this study looked at the effects of ivermectin in rabbits and the results should further our understanding of the potential toxicological effects of ivermectin and its wider application in the veterinary field.

## Material and methods

The study included 10 clinically healthy adult male rabbits (Oryctolagus cuniculus) from the age of 10 to 12 months, weighing 1.5 to 2 kg. The animals were kept in a physiology laboratory at the University of Fallujah under controlled climate conditions (23 ± 3°C, 12 hours of light and dark cycles, and 50-60 % relative humidity). They were provided with a balanced commercial diet and unrestricted access to water. Before the experimental methods, all animals underwent an acclimatization phase of two weeks to minimize handling stress and achieve full acclimatization to the laboratory environment.

After acclimation, rabbits were randomly allocated to one of two groups of five animals: a control group (no treatment) and a group ivermectin (Ivermac-10 ^®^, ADWIA Pharmaceuticals, Egypt) that received subcutaneous administration of 0.25 mg ivermectin once weekly for 4 weeks. Before sampling, animals were lightly anaesthetised with xylazine (6 mg per kg body weight, intramuscular) ( Sarwar et al., 2014) to reduce the discomfort of the procedure and blood samples were obtained by cardiac puncture.

All procedures in animals, including anaesthesia and blood sampling, were in accordance with the recommendations of the American Vets Medical Association ( Underwood et al., 2013). The blood samples were centrifuged at a rate of 3000 rpm for 10 minutes to obtain a serum sample which was then stored at -20-20°C for further investigation. Serum concentrations of sodium, potassium and chloride were measured with the using a Diasys Respons ^®^ 920 automatic analyser (Diasys Diagnostic Systems, Germany). Biochemical parameters for this study using colorimetric assay Kit (Agape Diagnostics Swtzerland for glucose, total protein, albumin and AST and ALT), (Sam Diagnostic, Dubai-UAE for urea, uric acid, cholesterol and superoxide dismutase activity) and (Biolabo SAS, France for creatinine, triglycerides, MDA and GSH) and (Elabscience, china for calcium, magnesium and phosphate). All data were statistically analyzed using a T-independent test (SPSS category 21) to demonstrate variations among groups. Values were described as means standard error at P ≤ 0.05 ( Larsen et al., 1973). All experimental procedures have been carried out in accordance with institutional guidelines on the care and use of laboratory animals.

## Results

### 1- Effect of Ivermectin on specific serum electrolyte ion values in rabbits

Ivermectin substantially increased (P ≤ 0.05) the potassium, sodium, chloride, and phosphate ion levels in blood serum compared to the control. Ivermectin did not affect serum calcium or magnesium concentrations ( Table 1).

**Table 1.** Effect of repeated administration of Ivermectin on specific serum electrolyte ion (K ^+^, Na ^+^, Cl ^- ^, Phosphate, Mg ^+2^, and Ca ^+2^) values in male rabbits.

Parameters	Control	Ivermectin
K ^+^ (mmol/L)	5.24 ± 0.23 ^B^	6.54 ± 0.2 ^A^
Na ^+^ (mmol/L)	143.4 ± 0.74 ^B^	158.4 ± 1.29 ^A^
Cl ^-^ (mmol/L)	112 ± 0.83 ^B^	117.4 ± 1.1 ^A^
Phosphate (U/L)	3.74 ± 0.27 ^B^	8.9 ± 0.63 ^A^
Mg ^+2^ (mg/dl)	2.23 ± 0.04 ^A^	2.3 ± 0.11 ^A^
Ca ^+2^ (mg/dl)	20.1 ± .73 ^A^	21 ± 1.17 ^A^

-Number of animals in each group (5), Data reflected as Mean ± Standard Error (SE).

-The different capital letters indicate significant changes at (P ≤ 0.05) group variation.

### 2- Effect of Ivermectin on specific serum biochemical values in rabbits

The effects of ivermectin treatment on the values of some serum biochemicals are shown in ( Table 2). All biochemical concentrations increased significantly (P ≤ 0.05) in the treatment groups compared to the control, except for a significant decrease in serum albumin concentration (P ≤ 0.05) and no significant changes in uric acid concentration.

**Table 2.** Effect of repeated administration of Ivermectin on specific serum biochemical (Glucose, Total protein, Albumin, Creatinine, Uric acid, Cholesterol, Triglyceride) values in male rabbits.

Parameters	Control	Ivermectin
Glucose mg/dl	52 ± 3.0 ^B^	75.6 ± 1.65 ^A^
Total protein g/dl	8.26 ± 0.17 ^B^	11.2 ± 0.55 ^A^
Albumin g/dl	5.3 ± 0.3 ^A^	3.4 ± 0.2 ^B^
Creatinine mg/dl	1.93 ± 0.25 ^B^	3.1 ± 0.29 ^A^
Uric acid mg/dl	8.3 ± 0.2 ^A^	8.24 ± 0.16 ^A^
Cholesterol mg/dl	44.4 ± 1.8 ^B^	69.8 ± 1.39 ^A^
Triglyceride mg/dl	46.2 ± 1.5 ^B^	72.4 ± 1.4 ^A^

-Number of animals in each group (5), Data reflected as Mean ± Standard Error (SE).

-The different capital letters indicate significant changes at (P ≤ 0.05) group variation.

### 3- Effect of Ivermectin on particular serum oxidative stress and liver enzymes (ALT and AST) values in rabbits

Table 3 shows that ivermectin administration resulted in a significant increase (P ≤ 0.05) in MDA, AST, and ALT levels, as well as a decrease in GSH and superoxide dismutase activity in serum compared to the control group.

**Table 3.** Effect of repeated administration of Ivermectin on specific serum oxidative parameters (malondialdehyde (MDA), reduced glutathione (GSH), Superoxide dismutase (SOD), alanine aminotransferase (ALT) and aspartate aminotransferase (AST)) values in male rabbits.

Parameters	Control	Ivermectin
MDA (μmol/l)	8.11 ± 0.31 ^B^	16.14 ± 0.8 ^A^
GSH (μmol/l)	14.2 ± 0.94 ^A^	8.4 ± 1.8 ^B^
SOD (U/ml)	12 ± 0.83 ^A^	7.4 ± 1.1 ^B^
AST (IU/l)	33.4 ± 0.49 ^B^	38.2 ± 0.57 ^A^
ALT (IU/l)	14 ± 0.86 ^B^	21.5 ± 0.93 ^A^

-Number of animals in each group (5), Data reflected as Mean ± Standard Error (SE).

-The different capital letters indicate significant changes at (P ≤ 0.05) variation between groups.

## Discussion

In rabbits, ivermectin induced multiorgan effects primarily via the liver and the kidney. Significant increases in serum sodium, potassium, chloride, and phosphate levels ( Table 1) indicate renal tubular dysfunction and electrolyte disturbances. As a highly lipophilic compound, ivermectin accumulates in the liver and kidney, where it interferes with cytochrome P450 enzymes and P-glycoprotein transporters ( Rendic, 2021), resulting in oxidative stress, tubular degeneration, and increased activity of hepatic enzymes ( El-Far, 2013; Salman et al., 2022; Miranda et al., 2025).

Significant increases in serum glucose, creatinine, triglycerides, cholesterol and total protein after repeated ivermectin administration ( Table 2) indicate concurrent liver and renal injury. Increase in creatinine, however, indicates impaired renal clearance. Adding insult to injury, hyperglycaemia and hyperlipidaemia indicate liver overload and disturbances of both carbohydrate and lipid metabolism ( Cao et al., 2025). Coinciding low serum albumin in ivermectin group indicates decreased hepatic synthesis capacity, which is consistent with the high liver enzymes observed below. In addition, metabolic disorders induced by ivermectin are likely mediated by mitochondrial and cellular ATP dependent blockade and by increased gluconeogenesis and lipolysis ( Wang et al., 2018).

In addition, ALT, AST and MDA levels increased significantly with a significant decrease in GSH and SOD activity ( Table 3), suggesting that ivermectin may be involved in oxidative stress as a major mechanism of cellular damage ( Wang et al., 2023). Increased MDA levels indicate increased lipid peroxidation, which reflects oxidative damage of the translational machinery of the cell. On the other hand, depletion of antioxidant parameters indicates that the innate protection against reactive oxygen species is depleted, leading to damage to the hepatocellular membrane and subsequent leakage of intracellular enzymes such as ALT and AST ( Abu Hafsa et al., 2021; Tang et al., 2022; El-Shobokshy et al., 2023). In addition, mitochondrial failure, combined with a decrease in ATP production, increases oxidative stress, which causes severe damage to both liver and kidney tissues ( Zhang et al., 2022).

The combination of prolonged oxidative stress and decreased liver and kidney function creates a self-sustaining imbalance that promotes deterioration. Secondary renal dysfunction occurs in association with abnormal storage of electrolytes, including sodium, potassium, chloride and phosphate ions. These changes are due to hepatic injury, reduced albumin synthesis and impaired glucose and lipid metabolism, all of which are due to long-term oxidative stress. This stress is due to accumulation of ivermectin and inhibition of cytochrome P450 enzymes, followed by excessive formation of ROS and eventual exhaustion of antioxidant protection. This pattern is consistent with previous studies in mammals of oxidative and metabolic stress induced by ivermectin ( Tawfeek et al., 2021; Salman et al., 2022; El-Shobokshy et al., 2023; Ali et al., 2025).

## Conclusion

In summary, this study explains the adverse effects of ivermectin in rabbits. Ivermectin induces oxidative stress and mitochondrial dysfunction in liver tissue, leading to biochemical disorders that extend to renal dysfunction and electrolyte imbalance. These findings suggest that even therapeutic or prolonged exposure may induce interrelated hepatic and renal dysfunction.

## Ethical considerations

This study was conducted in full compliance with the ethical guidelines approved by the Scientific Research Ethics Committee of the Faculty of Veterinary Medicine, University of Fallujah (Approval No. 6; 13/10/2025), https://doi.org/10.5281/zenodo.17832715. ( Saud et al., 2025).

## Data Availability

Underlying data Zenodo: Dataset for the Ivermectin Study in Rabbits (2025). https://doi.org/10.5281/zenodo.17832715.( Saud et al., 2025). **Arrive checklist**: https://doi.org/10.5281/zenodo.17832715 ( Saud et al., 2025). Data are available under the terms of the Creative Commons Attribution 4.0 International license (CC-BY 4.0).
