# Physiological and Biochemical Responses to Repeated Administration of Ivermectin in Rabbits

**DOI:** 10.12688/f1000research.174211.2

**Published:** 2026-06-01

**Authors:** Mustafa A. Saud, Sabea Khamees Abed, Mohammed Ali Shahooth, Mohammad Jadaan, Ahmed Emad Abood

**Affiliations:** 1 College of Veterinary Medicine, University of Fallujah, Al-Fallujah, Al Anbar Governorate, 00964, Iraq; 2 Anatomy, University of Anbar, Ramadi, Al Anbar Governorate, Iraq

**Keywords:** Ivermectin, Biochemical, Electrolytes, Oxidative Markers

## Abstract

**Background:**

While ivermectin is a common antiparasitic drug used in veterinary medicine, the effects of repeated administration at therapeutic doses have not been well studied. Of particular interest is the impact of ivermectin on electrolyte balance, blood biochemistry, and oxidation-related markers in rabbits, as these animals are known to be sensitive to drug-related metabolic disturbances and side effects.

**Methods:**

A total of 10 clinically healthy adult male rabbits (1.5-2 kg) were randomly assigned to a control group with no treatment or to a treatment group receiving ivermectin subcutaneously at a dose of 0.25 mg/kg once weekly for 30 days. The animals were housed in the physiological laboratory of the University of Fallujah under controlled environmental conditions. At the end of the experiment, blood samples were collected, and serum was analyzed for electrolytes, biochemical and oxidative markers, and liver enzyme activity using established laboratory methods.

**Results:**

Repeated administration of ivermectin caused a statistically significant increase in potassium, sodium, chloride, and phosphate concentrations (P≤0.05) compared with baseline, while calcium and magnesium levels remained unchanged. Glucose, total protein, creatinine, cholesterol, and triglyceride levels increased statistically significantly (P≤0.05) compared with the control group, while albumin concentration decreased. Oxidative stress indicators showed increased levels of malondialdehyde (MDA) and decreased levels of reduced glutathione (GSH), as well as superoxide dismutase (SOD) activity. Increased alanine aminotransferase (ALT) and aspartate aminotransferase (AST) activity were also noted.

**Conclusions:**

Repeated administration of ivermectin at therapeutic doses at short intervals causes disturbance in blood electrolyte balance, biochemical profile, and antioxidant system in rabbits, and may also negatively impact liver and kidney functions. These results indicate the need for cautions use of the drug, preferably limited to single administration at long intervals, to minimize the risk of side effects.

## Introduction

Ivermectin is a semisynthetic derivative of a vermectin, a naturally occurring macrocyclic lactone produced by the bacterium *Streptomyces avermitilus* ( Hazan, 2022). This lipophilic macrocytic lactone compound exhibits broad -spectrum antiparasitic activity and is widely employed in both veterinary and human healthcare to control nematodes, arthropods, and other parasitic infections ( Hasan et al., 2022). Ivermectin is commonly used in animal husbandry systems for the prevention and treatment of infections caused by internal parasites in horses, cattle, sheep and goats ( Parisi et al., 2019; Bordes et al., 2020; Ahmed et al., 2020). Due to its wide use, the physiological and pharmacological effects of ivermectin on target species remain the subject of ongoing research. Ivermectin is the only avermectin derivative authorised for use in humans and is effective against parasitic infections such as onchocerciasis and lymphatic filariasis. Its antiparasitic activity is primarily mediatrd through activation of glutamate-gated chloride channels allowing for continuous flow of chloride, blocking the transfer of signals from the central interneurons to peripheral mite neurons. Thus, membrane hyperpolarization reduces cellular excitability, and the parasite becomes paralyzed and eventually die ( Wolstenholme and Neveu, 2022: Shwaish et al., 2024).

Despite its proven efficacy and extensive concerns have been raised regarding potential adverse effects in treated animals. Ivermectin exhibits a variety of pharmacokinetic properties, including high lipid solubility, long half-life and extensive tissue distribution, especially in the liver and adipose tissue ( Hennessy and Alvinerie, 2002). These properties may cause bioaccumulation and delayed elimination, especially via milk excretion during lactation. Ivermectin is also extensively metabolised in the liver by cytochrome P450 (CYP450) enzymes. Experimental studies suggest that ivermectin and its metabolites may modulate the cytochrome P450 activity and related drug transporters in mammals, potentially contributing to altered hepatic metabolism and biochemical disturbances ( Salman et al., 2022).

Several studies have shown that avermectins (ivermectin, abamectin, doramectin and eprinomectin) may cause a variety of biochemical, hormonal and histopathological changes in animals exposed to these substances ( GabAllh et al., 2017; Salman et al., 2022). In cattle, prolonged or repeated dosing of these substances, even at therapeutic levels, has been associated with disturbances of endocrine and reproductive function. This includes dysregulation such as hormonal imbalances, altered reproductive patterns and reduced fertility ( Sadek and Shaheen, 2015; Nicolas et al., 2020).

Ivermectin is given routinely to rabbits to prevent and treat gastrointestinal parasitic infestations and mange ( Sharun et al., 2019). Rabbits are of economic importance as an agricultural resource, as well as a valuable model for biomedical research, and it is therefore imperative to maintain their physiological integrity by maintaining effective antiparasitic management protocols. But repeated or prolonged exposure to these compounds could lead to subclinical physiological changes which may adversely affect animal welfare and productivity. Therefore, the present study was designed to evaluate the effects of repeated ivermectin administration on biochemical and mineral parameters in rabbits to provide further insight.

## Material and methods

Ten clinically healthy adult male rabbits (Oryctolagus cuniculus) weighing 1.5 to 2 kg (aged 10 to 12 months) were included in the study. The animals were maintained in the physiology laboratory of the University of Fallujah under controlled climate conditions: 23 ± 3 °C, 12-hour light and dark periods, and a relative humidity of 50-60%.Food was fed daily using a commercial balanced diet and water was freely available. Before the initiation of the experimental procedures to minimize handling stress and achieve full acclimatization to the laboratory environment. to minimize handling stress and achieve full acclimatization to the laboratory environment.

After acclimation, rabbits were randomly allocated to one of two groups of five animals: a control group (no treatment) and an ivermectin-treated group (Ivermac-10 ®, ADWIA Pharmaceuticals, Egypt) that received ivermectin at a dose of 0.25 mg/kg body weight by subcutaneous injection once weekly for 4 weeks. Prior to blood collection, animals were sedated with xylazine (6 mg per kg body weight, intramuscular) ( Sarwar et al., 2014) to minimize procedural stress and facilitate blood collection by cardiac puncture.

All animal experimentation, including blood sampling or anaesthetic, have been carried out according to the ethical guidelines and recommendations of the American Veterinary Medical Association (AVMA) for proper care and treatment of experimental animals ( Underwood et al., 2013). Blood samples were centrifuged at 3000 rpm for 10 minutes and the serum was stored at −20 °C until biochemical analysis. The concentrations of serum sodium, potassium and chloride were determined by the automated biochemical analyzer DiaSys Respons 920, manufactured by DiaSys Diagnostic Systems, Germany, according to standard analysis method. Biochemical parameters were determined using colorimetric assay Kit (Agappe Diagnostics Swtzerland for glucose, total protein, albumin and AST and ALT), (Sam Diagnostic, Dubai-UAE for urea, uric acid, cholesterol and superoxide dismutase activity) and (Biolabo SAS, France for creatinine, triglycerides, MDA and GSH) and (Elabscience, China for calcium, magnesium and phosphate). Data was analyzed statistically using SPSS (version 21.0; IBM). Data are reported as mean ± SEM. The Shapiro-Wilk test was used to determine the significance. Independent two-sample t-test was used to evaluate differences between the occlusion and control groups. The false discovery rate (FDR) was controlled using the Benjamini-Hochberg correction and the significance level was set at p ≤ 0.05 to minimize the risk of type I errors due to multiple comparisons ( Larsen et al., 1973).

**Table 2.** Effect of repeated administration of Ivermectin on specific serum biochemical (Glucose, Total protein, Albumin, Creatinine, Uric acid, Cholesterol, Triglyceride) values in male rabbits.

Parameters	Control	Ivermectin	P-value	
Glucose mg/dl	52 ± 3.0 ^B^	75.6 ± 1.65 ^A^	0.00021	
Total protein g/dl	8.26 ± 0.17 ^B^	11.2 ± 0.55 ^A^	0.00082	
Albumin g/dl	5.3 ± 0.3 ^A^	3.4 ± 0.2 ^B^	0.00039	
Creatinine mg/dl	1.93 ± 0.25 ^B^	3.1 ± 0.29 ^A^	0.0116	
Uric acid mg/dl	8.3 ± 0.2 ^A^	8.24 ± 0.16 ^A^	0.73	
Cholesterol mg/dl	44.4 ± 1.8 ^B^	69.8 ± 1.39 ^A^	6.7 × 10 ^−6^	
Triglyceride mg/dl	46.2 ± 1.5 ^B^	72.4 ± 1.4 ^A^	2.8 × 10 ^−7^	

-Number of animals in each group (5), Data reflected as Mean ± Standard Error (SE).

-The different capital letters indicate significant changes at (P ≤ 0.05) group variation.

## Results

### 1- Effect of Ivermectin on specific serum electrolyte ion values in rabbits

Ivermectin substantially increased (P ≤ 0.05) the potassium, sodium, chloride, and phosphate ion levels in blood serum compared to the control. Ivermectin did not affect serum calcium or magnesium concentrations ( Table 1).

**Table 1.** Effect of repeated administration of Ivermectin on specific serum electrolyte ion (K ^+^, Na ^+^, Cl ^- ^, Phosphate, Mg ^+2^, and Ca ^+2^) values in male rabbits.

Parameters	Control	Ivermectin	P-value	
K ^+^ (mmol/L)	5.24 ± 0.23 ^B^	6.54 ± 0.2 ^A^	0.0035	
Na ^+^ (mmol/L)	143.4 ± 0.74 ^B^	158.4 ± 1.29 ^A^	0.0023	
Cl ^-^ (mmol/L)	112 ± 0.83 ^B^	117.4 ± 1.1 ^A^	0.0042	
Phosphate (U/L)	3.74 ± 0.27 ^B^	8.9 ± 0.63 ^A^	0.00031	
Mg ^+2^ (mg/dl)	2.23 ± 0.04 ^A^	2.3 ± 0.11 ^A^	0.32	
Ca ^+2^ (mg/dl)	20.1 ± .73 ^A^	21 ± 1.17 ^A^	0.58	

-Number of animals in each group (5), Data reflected as Mean ± Standard Error (SE).

-The different capital letters indicate significant changes at (P ≤ 0.05) group variation.

### 2- Effect of Ivermectin on specific serum biochemical values in rabbits

The effects of ivermectin treatment on the values of some serum biochemicals are shown in ( Table 2). All biochemical concentrations increased significantly (P ≤ 0.05) in the treatment groups compared to the control, except for a significant decrease in serum albumin concentration (P ≤ 0.05) and no significant changes in uric acid concentration.

### 3- Effect of Ivermectin on particular serum oxidative stress and liver enzymes (ALT and AST) values in rabbits

Table 3 shows that ivermectin administration resulted in a significant increase (P ≤ 0.05) in MDA, AST, and ALT levels, as well as a decrease in GSH and superoxide dismutase activity in serum compared to the control group.

**Table 3.** Effect of repeated administration of Ivermectin on specific serum oxidative parameters (malondialdehyde (MDA), reduced glutathione (GSH), Superoxide dismutase (SOD), alanine aminotransferase (ALT) and aspartate aminotransferase (AST)) values in male rabbits.

Parameters	Control	Ivermectin	P-value	
MDA (μmol/l)	8.11 ± 0.31 ^B^	16.14 ± 0.8 ^A^	1.2 × 10 ^−7^
GSH (μmol/l)	14.2 ± 0.94 ^A^	8.4 ± 1.8 ^B^	0.0003
SOD (U/ml)	12 ± 0.83 ^A^	7.4 ± 1.1 ^B^	0.0062
AST (IU/l)	33.4 ± 0.49 ^B^	38.2 ± 0.57 ^A^	4.6 × 10 ^−5^
ALT (IU/l)	14 ± 0.86 ^B^	21.5 ± 0.93 ^A^	3.5 × 10 ^−5^

-Number of animals in each group (5), Data reflected as Mean ± Standard Error (SE).

-The different capital letters indicate significant changes at (P ≤ 0.05) variation between groups.

## Discussion

Following repeated administration of ivermectin to rabbits, biochemical changes were observed may indicating liver and kidney dysfunction. Significant increases in serum sodium, potassium, chloride, and phosphate levels ( Table 1) may be due to changes in renal electrolyte handling and electrolyte disturbances. As a highly lipophilic compound, ivermectin accumulates in the liver and kidney, it can change the activity of xenobiotic metabolizing enzymes and transporters, which can result in changes to cellular homeostasis ( Rendic, 2021), This can lead to oxidative imbalance and changes in biochemical processes ( El-Far, 2013; Salman et al., 2022; Miranda et al., 2025).

Significant increases in serum glucose, creatinine, triglycerides, cholesterol and total protein after repeated ivermectin administration ( Table 2) suggest possible hepatic and renal functional alterations. Increase in creatinine, however, indicates impaired renal clearance. Furthermore, hyperglycaemia and hyperlipidaemia may reflect disturbances in metabolic regulation and disturbances of both carbohydrate and lipid metabolism ( Abed and Al-Azawi, 2020; Cao et al., 2025). Reduced serum albumin may impaired hepatic synthetic function, which is consistent with the high liver enzymes observed below. In addition, metabolic disorders induced by ivermectin are possibly associated with altered cellular energy metabolism and by increased gluconeogenesis and lipolysis ( Wang et al., 2018).

In addition, ALT, AST and MDA levels increased significantly with a significant decrease in GSH and SOD activity ( Table 3), suggesting that ivermectin may be involved in oxidative stress as a major mechanism of cellular damage ( Wang et al., 2023). Increased MDA levels indicate increased lipid peroxidation, which reflects oxidative damage to membrane lipids. On the other hand, depletion of antioxidant parameters indicates that the endogenous antioxidant defenses may be compromised, potentially contributing to hepatocellular membrane instability and subsequent leakage of intracellular enzymes such as ALT and AST ( Abu Hafsa et al., 2021; Tang et al., 2022; El-Shobokshy et al., 2023). In addition, mitochondrial dysfunction, combined with a decrease in ATP production, increases oxidative stress, which may contribute to fuctional disturbances in hepatic and renal tissues ( Zhang et al., 2022).

The combination of prolonged oxidative stress and decreased liver and kidney function may contribute to progressive metabolic dysregulation. Electrolyte imbalance may reflect altered renal excretion or redistribution, including sodium, potassium, chloride and phosphate ions. These alterations may be associated with metabolic disturbances involving hepatic and renal function are due to hepatic injury, reduced albumin synthesis and impaired glucose and lipid metabolism, all of which are due to long-term oxidative stress. This oxidative imbalance may be related to repeated ivermectin exposure, followed by excessive formation of ROS and eventual exhaustion of antioxidant protection. This pattern is consistent with previous studies in mammals of oxidative and metabolic stress induced by ivermectin ( Tawfeek et al., 2021; Salman et al., 2022; El-Shobokshy et al., 2023; Ali et al., 2025).

## Conclusion

In summary, this study demonstrates biochemical alterations associated with repeated ivermectin exposure in rabbits. Repeated ivermectin exposure may be associated with oxidative stress and possible mitochondrial, which may contribute to biochemical disturbances, altered renal function, and electrolyte imbalance. These findings suggest that even therapeutic or prolonged exposure may be associated with concurrent hepatic and renal functional disturbances.

## Ethical considerations

This study was conducted in full compliance with the ethical guidelines approved by the Scientific Research Ethics Committee of the Faculty of Veterinary Medicine, University of Fallujah (Approval No. 6; 13/10/2025), https://doi.org/10.5281/zenodo.17832715. ( Saud et al., 2025).

## Data Availability

Underlying data Zenodo: Dataset for the Ivermectin Study in Rabbits (2025). https://doi.org/10.5281/zenodo.17832715.( Saud et al., 2025). **Arrive checklist**: https://doi.org/10.5281/zenodo.17832715 ( Saud et al., 2025). Data are available under the terms of the Creative Commons Attribution 4.0 International license (CC-BY 4.0).
