# Supplementary material for: Physiological and Biochemical Responses to Repeated Administration of Ivermectin in Rabbits
Source: F1000Res. 2026 Jun 1;15:225. Originally published 2026 Feb 10. [Version 2] doi: 10.12688/f1000research.174211.2 (PMC13287982; doi:10.12688/f1000research.174211.2)
Supplement: Peer review history

# Reviewer report: Reviewer response for version 2

**DOI:** 10.5256/f1000research.202254.r491108

**Published:** 2026-06-23

**Authors:** Dina S Dheyab

**Affiliations:** 1 University of Baghdad, Baghdad, Iraq

**The research is very good scientifically, but some aspects need strengthening the language with certain terminology. I recommend accepting the research. Thank you.**

**Strengths of the study**

1. The study investigates an important issue: **repeated administration of ivermectin**, not just a single dose.
2. It includes a **wide range of parameters**, such as:
  - Electrolytes
  - Biochemical profile
  - Oxidative stress markers
  - Liver enzyme activity
3. The dose used (0.25 mg/kg) is within a **therapeutic range**, making the findings more relevant to real veterinary practice.

**Methodological and scientific limitations**

**1. Very small sample size**

- Only **10 rabbits** were used in total.
- This greatly reduces **statistical power** and increases the risk that results are influenced by individual variability.

**2. Lack of intermediate measurements**

- Blood samples were taken only at the end of the experiment.
- Therefore, it is unclear:
  - When the changes started
  - Whether they were progressive or temporary
  - Whether the effects were reversible

**3. Short duration of the study**

- The experiment lasted only **30 days**.
- This is insufficient to assess **long-term or chronic toxicity**.

**4. No histopathological examination**

- The study suggests possible liver and kidney damage based on ALT, AST, and creatinine levels.
- However, there is **no tissue-level confirmation** (no liver or kidney histology

**5.Weak explanation of electrolyte disturbances**

- Significant changes in Na⁺, K⁺, Cl⁻, and phosphate were reported.
- However, the study provides **limited mechanistic explanation** for these disturbances.

**6.No dose–response design**

- Only one dose level was tested.

Is the work clearly and accurately presented and does it cite the current literature?

Yes

If applicable, is the statistical analysis and its interpretation appropriate?

Yes

Are all the source data underlying the results available to ensure full reproducibility?

Yes

Is the study design appropriate and is the work technically sound?

Yes

Are the conclusions drawn adequately supported by the results?

Yes

Are sufficient details of methods and analysis provided to allow replication by others?

Yes

Reviewer Expertise:

endocrine disorders single-cell technologies

I confirm that I have read this submission and believe that I have an appropriate level of expertise to confirm that it is of an acceptable scientific standard.

# Reviewer report: Reviewer response for version 2

**DOI:** 10.5256/f1000research.202254.r491113

**Published:** 2026-06-23

**Authors:** Ali Mosa Rashid Al-Yasari

**Affiliations:** 1 Al-Muthanna University, Samawah, Iraq

**Your Report**

 General Evaluation

 The manuscript investigates the physiological, biochemical, and oxidative stress impacts of repeated therapeutic subcutaneous administrations of ivermectin (0.25 mg/kg} weekly for 4 weeks) in male rabbits. Given the ubiquitous use of ivermectin in veterinary medicine for treating conditions like sarcoptic mange, evaluating subclinical toxicities associated with repeated exposure is highly relevant.  

 The authors have visibly addressed several structural shortcomings from Version 1 by organizing the methodology with better kit descriptions and clarifying group numbers (n=5). However, critical scientific gaps, contradictions within the text, and reporting vulnerabilities remain in this revised version. Notably, the manuscript still claims to evaluate "physiological" responses, yet it only reports serum biochemical data. Additionally, there are major discrepancies in statistical parameter reporting and questionable physiological baselines that require thorough rectification before approval.  

 Major Comments

 1. Misalignment of Title/Objectives with Results (Missing Physiological Data):

 The title and objectives explicitly promise an evaluation of physiological responses. True physiological data typically include clinical signs, body weight changes, feed/water intake, fecal output, or vital signs (temperature, heart rate, respiration rate). The results presented are exclusively biochemical endpoint markers from serum. The authors must either supply the missing clinical/physiological data or remove "Physiological" from the title and text to avoid misleading the reader.  

 2. Inconsistencies in Methodological Terminology & Blinding:

  In the Material and Methods section, the authors state: "Independent two-sample t-test was used to evaluate differences between the occlusion and control groups." The term "occlusion group" is highly confusing contextually. It appears to be a copy-paste or translation error for the "ivermectin-treated group". This must be corrected to maintain professional clarity.  

  There is no mention of whether sample analysis or data handling was blinded. Given the small sample size (n=5 per group), bias control is crucial.  

 3. Statistical Reporting and Logical Errors:

 4. Biochemical and Physiological Implausibility:

 If total protein rises drastically while albumin plummets, it implies an immense, unaddressed spike in the globulin fraction. The authors completely ignore this dynamic in the discussion. What is driving this massive globulin increase? Is it an acute phase inflammatory response, or severe hemoconcentration/dehydration? This needs profound biochemical interpretation.  

 Minor Comments

 1. Typographical and Grammatical Errors:

  Material and Methods (Paragraph 1): The sentence fragment "...to minimize handling stress and achieve full acclimatization to the laboratory environment." is accidentally duplicated back-to-back at the end of the paragraph. Please excise the duplicate text.  

  Introduction (Paragraph 1): Typo in "Its antiparasitic activity is primarily mediatrd through...". Correct "mediatrd" to "mediated".  

  Discussion (Paragraph 2): Grammatical error in "Reduced serum albumin may impaired hepatic synthetic function...". Change to "Reduced serum albumin may indicate impaired hepatic synthetic function..." or "...may impair...".  

 2. Incomplete Manufacturer Data:

 In the Material and Methods section, the colorimetric kits are cited with manufacturers and cities. Please provide the full formal corporate names, specific catalog numbers if available, and complete locations (City, Country) for all diagnostic test kits to maximize transparency and reproducibility.  

 3. Units Format Consistency:

 Ensure standard scientific formatting is used uniformly across all tables. 

 Conclusion

 This manuscript provides practical insights into the subclinical biochemistry profiles of ivermectin overuse. However, it requires a Moderate-to-Major Revision to eliminate textual errors, reconcile highly unusual reference baselines, and properly align the stated physiological scope with the purely biochemical results presented.

Is the work clearly and accurately presented and does it cite the current literature?

Partly

If applicable, is the statistical analysis and its interpretation appropriate?

Partly

Are all the source data underlying the results available to ensure full reproducibility?

Partly

Is the study design appropriate and is the work technically sound?

Yes

Are the conclusions drawn adequately supported by the results?

No

Are sufficient details of methods and analysis provided to allow replication by others?

Partly

Reviewer Expertise:

Biochemistry

I confirm that I have read this submission and believe that I have an appropriate level of expertise to confirm that it is of an acceptable scientific standard, however I have significant reservations, as outlined above.

# Reviewer report: Reviewer response for version 2

**DOI:** 10.5256/f1000research.202254.r491109

**Published:** 2026-06-15

**Authors:** Mohammed Hayder Asker

**Affiliations:** 1 Mustansiriyah University, Baghdad, Iraq

Peer Review Report

 Summary of the Article

 The manuscript entitled “Physiological and Biochemical Responses to Repeated Administration of Ivermectin in Rabbits” investigates the physiological and biochemical effects associated with repeated ivermectin administration in rabbits. The study evaluates changes in selected hematological, biochemical, and physiological parameters to assess the potential impact and safety profile of repeated ivermectin exposure. The topic is relevant due to the widespread use of ivermectin in veterinary medicine and the need to better understand its effects following repeated administration.

 Overall Assessment

 The manuscript addresses an important veterinary and pharmacological topic. The study is generally well designed, and the findings contribute useful information regarding the physiological and biochemical responses of rabbits to repeated ivermectin treatment. The manuscript is clearly written and presents results that may be valuable for researchers and veterinary practitioners.

 Major Comments

 The study addresses a relevant scientific question and provides data that may contribute to understanding the safety and biological effects of repeated ivermectin administration.

 The experimental design appears appropriate for the objectives of the study. However, the authors may further strengthen the manuscript by providing additional discussion comparing their findings with those reported in previous studies.

 The discussion section could be expanded to better explain the physiological mechanisms responsible for the observed changes in the measured parameters.

 The authors should clearly highlight the practical implications of their findings for veterinary practice and future research.

 Minor Comments

 Some grammatical and stylistic revisions would improve readability.

 The manuscript would benefit from a more detailed description of certain methodological procedures to enhance reproducibility.

 Ensure that all abbreviations are defined at their first appearance in the text.

 A careful review of formatting and reference style is recommended.

 Strengths

 The study investigates a topic of practical veterinary importance.

 Appropriate physiological and biochemical parameters were evaluated.

 The manuscript is generally well organized and easy to follow.

 The findings provide useful information regarding repeated ivermectin administration in rabbits.

 Weaknesses

 The discussion could provide deeper interpretation of the biological significance of the findings.

 Additional comparison with recent literature would strengthen the conclusions.

 Some methodological details may require further clarification.

 Recommendation

 Approved

 The manuscript is scientifically sound and suitable for indexing after consideration of the minor comments above.

Is the work clearly and accurately presented and does it cite the current literature?

Yes

If applicable, is the statistical analysis and its interpretation appropriate?

Yes

Are all the source data underlying the results available to ensure full reproducibility?

Yes

Is the study design appropriate and is the work technically sound?

Yes

Are the conclusions drawn adequately supported by the results?

Yes

Are sufficient details of methods and analysis provided to allow replication by others?

Yes

Reviewer Expertise:

animal physiology

I confirm that I have read this submission and believe that I have an appropriate level of expertise to confirm that it is of an acceptable scientific standard.

# Reviewer report: Reviewer response for version 1

**DOI:** 10.5256/f1000research.192094.r470683

**Published:** 2026-04-08

**Authors:** Oscar Vivanco-Galván

**Affiliations:** 1 Universidad Técnica Particular de Loja, San Cayetano Alto, Ecuador

**General Comments**

 The manuscript addresses an interesting and relevant topic, particularly considering the widespread use of ivermectin as a broad-spectrum antiparasitic agent in animals. However, the potential effects of continuous or repeated dosing on organismal health remain insufficiently understood, which makes this study potentially valuable.

 Despite this, the manuscript requires substantial improvement in terms of clarity, structure, methodological detail, and scientific depth before it can be considered for publication.

**Specific Comments**

**Introduction**

 The Introduction is currently too general and lacks sufficient focus on the specific research problem. The authors are encouraged to:

- Expand the background on the **use of ivermectin in rabbits**, highlighting existing knowledge gaps.
- Provide a more detailed overview of the **physiological and biochemical effects of ivermectin**, including known dose-dependent responses.
- Ensure alignment between the **title, objectives, and methodological approach**, particularly regarding physiological and biochemical evaluations.
- Clearly and explicitly state the **objectives and/or hypotheses** of the study.

**Methodology**

 The Methods section lacks clarity and organization and should be substantially revised:

- Structure the section using clear subheadings, such as:
  - *Biological material*
  - *Experimental design*
  - *Experimental conditions*
  - *Statistical analysis*
- The manuscript does not specify the **number of animals used** in the experimental and control groups, which is a critical omission.
- The description of procedures should be improved for clarity and correctness. For example:
  “The blood samples were centrifuged at 3000 rpm for 10 minutes to obtain serum, which was subsequently stored at −20 °C until analysis.”

**Results**

 The study claims to evaluate **physiological responses**, yet these data are not clearly presented or are missing. The authors should:

- Ensure that all variables described in the objectives and methods are **fully reported in the Results section**.
- Present physiological data (if collected) in a clear and structured manner, including appropriate statistical analysis.

**Discussion**

 The Discussion requires significant improvement and deeper scientific interpretation:

- The section currently focuses mainly on reporting results rather than interpreting them in the context of existing literature.
- The authors should incorporate and compare their findings with **previous studies in other experimental species**.
- Provide a structured discussion addressing:
  - **Serum electrolyte levels**
  - **Biochemical parameters**
  - **Oxidative stress markers**
  - **Liver enzyme activity**
- Each variable should be discussed in relation to known physiological mechanisms and potential effects of ivermectin.

**Final Recommendation**

 The manuscript has potential but requires **major revision**. The authors should:

- Improve the overall quality of scientific writing.
- Provide missing methodological details.
- Strengthen the Discussion with critical analysis and literature support.
- Include additional relevant references to contextualize the findings.

Only after substantial revision and clarification of these aspects can the manuscript be adequately evaluated for indexing.

Reviewer Expertise:

NA

I confirm that I have read this submission and believe that I have an appropriate level of expertise to confirm that it is of an acceptable scientific standard, however I have significant reservations, as outlined above.

# Response

**Published:** 2026-05-21

**Authors:** Sabea Abed

**Affiliations:** veterinary medicine, University of Fallujah, Al-Fallujah, Al Anbar Governorate, Iraq

Dear Oscar Vivanco-Galván,

 We sincerely appreciate your valuable comments and constructive suggestions regarding our manuscript. All comments have been carefully considered, and substantial revisions have been made throughout the manuscript to improve its scientific quality, clarity, organization, and methodological accuracy.

**The Introduction** section has been extensively revised to provide a more focused background on ivermectin use in rabbits, its physiological and biochemical effects, existing knowledge gaps, and the specific objectives of the study.

**The Materials and Methods** section has been reorganized using clear subheadings, including biological materials, experimental design, experimental conditions, and statistical analysis. Additional methodological details were added, including the number of animals in each group, sample processing procedures, and statistical methods.

**The Results section** has been revised to ensure that all measured variables are clearly presented with appropriate statistical analysis and improved table organization.

**The Discussion section** has been substantially expanded and restructured to provide deeper scientific interpretation of the findings in relation to previous studies and known physiological mechanisms, particularly regarding electrolyte disturbances, biochemical alterations, oxidative stress markers, and liver enzyme activity.

 In addition, the manuscript has undergone comprehensive language editing and scientific revision to improve clarity and consistency throughout the text.

 We are grateful for your insightful comments, which significantly improved the quality of the manuscript.

 Sincerely        ,

 Sabea Khamees Abed
